# Pharmaceutical Wastewater as an Emerging Environmental Contaminant: Sustainable Treatment Strategies and Future Perspectives

**DOI:** 10.3390/bioengineering13050540

**Published:** 2026-05-07

**Authors:** Dhananjay Singh, Jyoti Kushwaha, Ravi Shankar, Sunita Singh, Vinay Mishra, Deepak Singh, Anshuman Mishra, Reeta Rani Singhania, Anil Kumar Patel, Balendu Shekher Giri

**Affiliations:** 1 Department of Chemical Engineering, Institute of Engineering & Technology, Lucknow 226021, UP, India; 2 Rajkiya Engineering College, Ambedkar Nagar, Akbarpur 210201, UP, India; 3 Department of Chemical Engineering, Madan Mohan Malviya University of Technology, Gorakhpur 273010, UP, Indiavinay.mishraa@gmail.com (V.M.); 4 Department of Pharmacy, Ambekeshwar Institute of Pharmaceutical Sciences, Lucknow 273010, UP, India; sunita.pharma80@gmail.com; 5 Department of Chemical Engineering, Bipin Tripathi Kumaon Institute of Technology, Dwarahat 263653, UK, India; 6 Institute of Aquatic Science and Technology, National Kaohsiung University of Science and Technology, Kaohsiung City 81157, Taiwan; 7 Department of Microbiology and Bioinformatics, Atal Bihari Vajpayee Vishvavidyalaya, Bilaspur 495009, CG, India; anilkpatel22@gmail.com; 8 Sustainability Cluster, University of Petroleum & Energy Studies, Dehradun 248007, UK, India

**Keywords:** pharmaceutical contaminants, water treatment, biological techniques, advanced oxidation process, hybrid methods

## Abstract

The level of pharmaceutical contaminants is increasing exponentially on planet Earth. Despite the vital role of medicines in life, pharmaceutical effluents have severe environmental impacts and cause health issues. In order to treat pharmaceutical effluents, a variety of methods are adopted globally. The conventional techniques lack the capability of effective removal of these hazardous effluents. This review focuses on the methods currently used to treat pharmaceutical wastewater. Both individual and hybrid treatment approaches have been investigated. Optimum and sustainable treatment methods have been presented. Their advantages and limitations have been discussed in detail. Modern treatment techniques are designed to be more sustainable and cost-effective, with a target to achieve high to near-complete removal of contaminants. No single technique is sufficient individually for the purpose. A suitable combination of biological treatment processes with a membrane system and advanced oxidation processes has been observed to be a highly effective method. However, such hybrid methods are designed according to the quality and quantity of wastewater, target pollutants, and several other crucial parameters.

## 1. Introduction

Pharmaceuticals are natural or synthetic chemical substances developed for specific purposes. Recently, the pharmaceutical industry has grown rapidly, and global antibiotic consumption has increased to 42–45 billion daily defined dose (DDD) in 2020–21 [1]. In the pharmaceutical industry, water is used in several forms at various stages, e.g., raw material, processing, and production [2,3]. An estimated 23 percent of water is utilized in the operating process, which leads to the production of highly harmful and contaminated effluents. Wastewater from various sources contains several pharmaceutical contaminants. These contaminants include antibiotics, nonsteroidal anti-inflammatory drugs (NSAIDs), analgesics, endocrine-disrupting compounds (EDCs), synthetic hormones and related chemicals, antiseptics, anti-epileptics, beta-blockers, anti-hypertensive medicines, and contraceptives [4,5]. Therefore, rigorous multistage treatment is required so as to meet regulatory requirements, which is very costly [6,7].

Pharmaceutical contaminants are a matter of rising concern as they affect both human and other ecological counterparts [8,9]. Assessing the methods and locations of pharmaceutical waste disposal is crucial, as improper handling of these by-products occurs frequently and poses significant risks [10]. High levels of ammonia, nitrogen, organic and inorganic compounds, and nutrients are found in pharmaceutical wastewater, resulting in elevated levels of chemical oxygen demand (COD) and biochemical oxygen demand (BOD). Various studies indicate the existence of approximately 200 to 600 different contaminants in wastewater [4,11]. Even a small quantity of these substances may have an adverse effect on the ecosystem. In water bodies, remnants of pharmaceutical substances are detected in ppm (mg/L) to ppb (μg/L) globally [12].

The presence of Gemfibrozil in industrial wastewater was reported to range from 3.47 to 63.8 mg/L [13]. Aspirin emerged as the most prevalent pharmaceutical compound in industrial wastewater samples, with a dose of 1180.82 mg/L. In contrast, nalidixic acid was identified as the most frequently detected antibiotic in the waste with a dose of 25.3 to 29.9 mg/L [14]. According to Phonsiri et al. [15], sulfamethoxazole, triclosan, erythromycin, and trimethoprim constituted a major part of influents in Southern California. Ciproxacin and sulfamethoxazole were the most commonly found contaminants in industrial wastewater [16]. Hospitals also make a significant contribution to wastewater pollution [17]. Water consumption in hospitals varies between 400 and 1200 L/bed/day depending upon location, nature, and size of the hospital. This leads to wastewater quantity reaching 200–1200 L/bed/day [18,19].

There are several reported adverse ecological effects of pharmaceutical contaminants. The presence of estrogen in aquatic environments may negatively impact male fertility [20,21]. These factors also have a detrimental impact on the health of newborns and elderly individuals, significantly exacerbating problems for those experiencing kidney and liver failure [22,23]. The presence of these pollutants may also elevate the likelihood of developing breast and testicular cancer. Presence of cancer-preventing drugs in water reservoirs may also impact the health of pregnant women, as these substances may significantly reduce cell viability of the human placenta and cause cytotoxicity along with endocrine dysfunction [24,25]. Continuous exposure to antibiotics may affect the development of anti-drug genes, thereby diminishing the effectiveness of infection treatment in both human beings and other creatures [26,27].

Therefore, the removal of pharmaceutical contaminants from wastewater is very crucial. Removal refers to the apparent elimination of a contaminant from the aqueous phase, which may occur via adsorption, separation, or partial transformation. However, this does not necessarily imply complete elimination of the compound, as it may persist in another phase or as transformation products. On the other hand, mineralization denotes its complete conversion into inorganic end products, e.g., CO_2_ and H_2_O. While many treatment processes achieve high removal efficiencies, complete mineralization is rare. Further, degradation often involves the alteration of the molecular structure, leading to transformation products rather than full mineralization into CO_2_, H_2_O, and inorganic ions.

Therefore, while advanced treatment methods can significantly reduce the concentration of target pharmaceutical contaminants, they frequently yield intermediate byproducts, whose persistence and potential toxicity remain a crucial issue. An optimum treatment method must also focus on the intermediates produced, in addition to the parent compounds, so as to ensure effective and sustainable remediation. Conventional biological, physical and chemical techniques are not capable of treating the wastewater up to the desired level [28,29]. Therefore, hybrid or advanced technologies are found to be suitable for the thorough eradication of pharmaceutical pollutants [30,31,32]. Hybrid technologies refer to a suitable combination of two or more conventional techniques [7]. This work reviews the physical, biological, and advanced oxidation processes (AOP) in detail, along with their advantages and limitations, with special attention to pharmaceutical wastewater treatment. Further, the hybrid or advanced methods are also presented.

## 2. Sources of Pharmaceutical Contaminants

**Figure 1:**
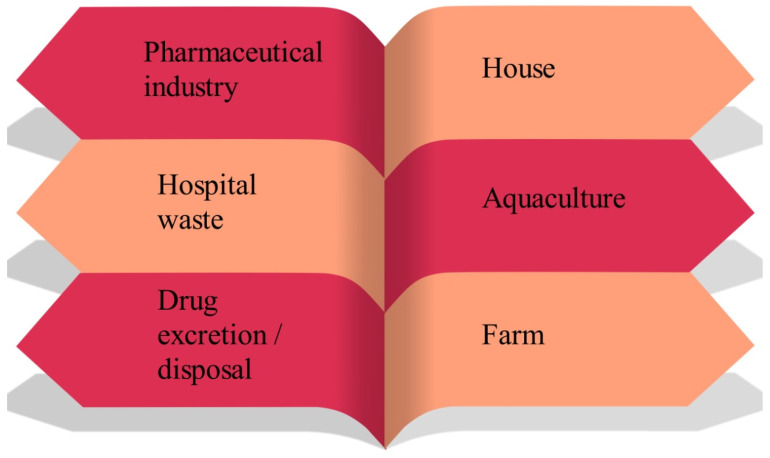
Sources of pharmaceutical contaminants.

Pharmaceutical products play a significant role in human life. Humans and animals generally consume these to treat their sicknesses and diseases. Pharmaceuticals are developed from organic materials in combination with additional materials for better performance. Pharmaceutical effluents can be classified either based on molecular weight (high or low) or solubility [33,34]. However, inappropriate disposal of these can severely affect human health and the environment [35,36]. Pharmaceutical compounds enter the environment via several routes, e.g., wastewater treatment plants (WWTP), landfills, hospitals, pharmaceutical manufacturing units, graveyards, etc. [10]. Pharmaceutical components may enter the aquatic environment using several pathways, e.g., inappropriate disposal, contaminated water discharge, and discharge of insufficiently treated wastewater [37,38]. Approximately 2000 types of pharmaceutical compounds are found worldwide [39]. Various sources of wastewater generation via pharmaceutical activities are shown in Figure 1.

### 2.1. Pharmaceuticals Related to Human

Numerous types of medicines are used by humans to prevent or treat diseases [40]. Global usage of antibiotics is found to be around 2 × 108 kg [41]. Various types of NSAIDs, e.g., analgesics, antipyretics, etc., are used in huge amounts globally. Anticonvulsants are used to treat epilepsy, seizure disorders, mental disorders, and neuralgia [42]. They get discharged into the environment and affect the ecology. The presence of excess amounts of human medication in the environment and water bodies causes severe problems for human health and animals. Sources of these drugs are healthcare clinics and related facilities, homes, and hospitals. Humans also contribute to the discharge of pharmaceutical effluents through urine or excretion [43]. Hospitals also dispose of medicines through drains, but still, the human contribution is more significant. Analysis done on the water revealed that human contribution is more than 90% [44]. Finally, pharmaceutical effluents enter water bodies, drinking water, and groundwater due to the incapability of the treatment techniques to eliminate the effluents effectively [45].

### 2.2. Pharmaceuticals Related to Animals

Many dedicated medicines are used to treat animal diseases. Veterinarians use antibiotics and NSAIDs to treat animal infections and other diseases [46]. Several antibiotics are added to animal feed so as to improve growth rate and also for prophylactic and chemotherapeutic purposes [47]. Pharmaceutical products related to animals can enter the environment through animal husbandry and horticulture, waste disposal, sewage effluent, and aquaculture [4,24,48]. Animals are usually incapable of absorbing the antibiotics completely. This leads to the excretion of a major amount of antibiotics through urine and excreta [49]. Also, animal excreta are used as fertilizer for crops, which increases the chances of contaminating the environment with antibiotic residues [50]. Animal husbandry operations like manure storage lagoons, farm field manure, aquaculture operations, dust, and farm field runoff are major operations responsible for water contamination with pharmaceutical impurities. Sufficient data is not available for the assessment of pharmaceutical effluents from animal origin.

## 3. Effects of Pharmaceutical Contaminants

The formulation of pharmaceutical substances is directed towards specific molecular and metabolic pathways [51]. Their consistent exposure may lead to bioaccumulation. Presence of drug contaminants (even in lower concentrations, 1–500 ng/L) in wastewater can affect aquatic life [52]. Diverse organisms react to these pharmaceutical substances in various ways [53,54]. Evidence suggests that pharmaceutical compounds have both direct and indirect adverse effects on various aquatic species, including fish. Diclofenac is observed to induce mortality in chickens and pigeons at concentration levels of 0.25 and 2.5 mg/kg, respectively [52]. As humans also consume aquatic animals, they also get exposed to these compounds. Pharmaceutical toxins also exist in aquatic ecosystems, where they are physiologically active and can affect the aquatic ecology [55].

Pharmaceutical contaminants affect humans, animals, and microorganisms in several ways, and these effects can be mild or severe depending on the type and quantity of the pharmaceutical compound. Antibiotics develop and transmit antibiotic-resistant genes in microorganisms [37,56]. Brain post-mortem examination confirms bioaccumulation of several pharmaceutical compounds in white matter brain tissues and hypothalamus [57]. Tetracycline residue in the aqueous environment can affect the human digestive system and also deter the proliferation of plants [58]. The occurrence of pharmaceutical pollutants results in both bioaccumulation and biomagnification throughout the aquatic food web [59]. The behaviour and feeding rate of wild fish, specifically Perca fluviatilis, were affected by exposure to oxazepam [4].

The detrimental impact on aquatic organisms can be linked to the bioaccumulation traits, persistence, and potential toxicity of pharmaceutical contaminants in their environment. The growth and development of microorganisms were also affected by pharmaceutical effluents [60]. Pharmaceutical effluents cause oxidative stress, leading to hepatocellular damage in fish [37]. Humans are also affected by the contamination due to pharmaceutical waste. Recent studies indicate that ibuprofen may adversely affect testicular and testosterone functions, resulting in a decrease in total testosterone levels in women, which is associated with the development of polycystic ovary syndrome [4,61]. Several bacterial species find the existence of triclosan to be toxic, while an abundance of alkyl benzene sulfonate alters microbial communities present in surface water [62].

**Figure 2:**
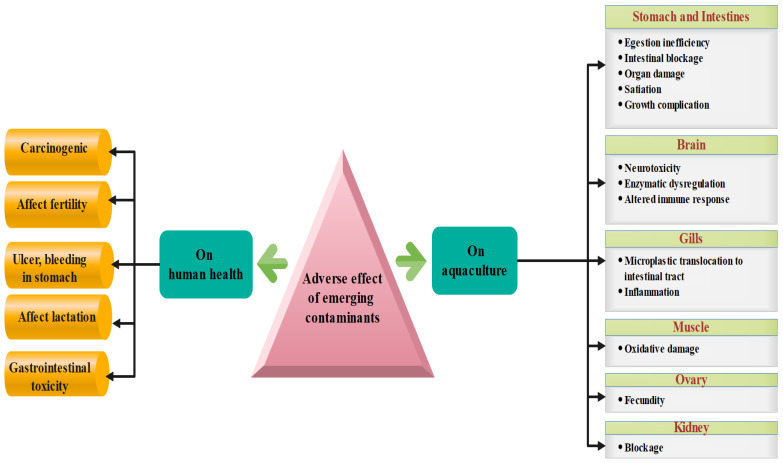
Adverse effects of pharmaceutical contaminants.

Pharmaceutical contaminants also pollute soil and endanger plants. Exposure of cucumber plants to pharmaceutical compounds resulted in stress indication and detoxification mechanisms [63]. Various pharmaceutical contaminants present in different concentrations affect the plants differently. It may induce a reduction in the lipid content, shifts in the metabolic reaction rates, an elevation in detoxification rates, and modifications to transpiration rates [64]. Sun et al. [65] testified to a decline in Hordeum distichum plant growth due to the presence of sulfamethazine (0.3–0.900 mg/L). A combination of 17 pharmaceutical contaminants having a total concentration of 550 μg/L caused a reduction in photosynthesis pigments in cucumber seedlings due to high accumulation of pharmaceutical compounds in the root areas. Major adverse effects of pharmaceutical contaminants on human health and aquaculture are shown in Figure 2. Due to these detrimental effects, the treatment of wastewater containing pharmaceutical contaminants becomes essential. Different strategies have been adopted for this purpose, and further research is in progress so as to optimize their effectiveness.

## 4. Wastewater Treatment

Conventional wastewater treatment techniques are more focused on the removal of macro-pollutants consisting of suspended solids, nitrogen, phosphorus, organic carbon, organic inorganic compounds, and pathogens instead of micro-pollutants [30,60]. The elimination efficacy depends upon various factors, e.g., contaminant flow rate, compound properties (size, functional group, concentration, and polarity), environmental conditions, and operating conditions [66]. Relevant literature indicates the use of multiple biological, physical, chemical, and hybrid techniques for the removal of pharmaceutical pollutants from the aqueous environment [38,67]. The typical wastewater treatment process entails the exposure of effluents to multiple physical/chemical/biological processes, including mineralization, dispersion, volatilization, dilution, and photo-degradation. Key processes in this context are the biodegradation and biotransformation carried out by microorganisms, alongside the sorption of materials onto biosolids [30].

High concentrations of several pharmaceutical contaminants were found at the discharge outlet of various WWTPs, indicating the incapability of conventional techniques to remove them efficiently [68]. Several wastewater treatment techniques used by various researchers for the removal of various contaminants are listed in Table 1.

### 4.1. Physical Processes for Removal of Pharmaceutical Contaminants

Pharmaceutical contaminants can be removed from wastewater using conventional physical techniques like sorption, coagulation-flocculation, sedimentation, and membrane filtration [97,98].

#### 4.1.1. Adsorption

One of the most common physical treatment strategies is adsorption. Activated carbon (AC) is the most common adsorbent used in the adsorption method for water cleaning [99]. It consists of carbonaceous matter and has a high surface area, porosity, and amorphous structure. Activated carbon can be prepared through an activation process at high temperatures using suitable biomass [100]. It can exist in microporous, macroporous, and mesoporous forms [101]. It is evident that removal of contaminants from water depends upon the contact time, surface area and other operating parameters.

It was found that contact time has a major impact on the number of pharmaceutical compounds absorbed by activated carbon. Powdered or granular activated carbon both have the same capability of treating pharmaceutical effluents [102,103]. Activated carbon shows higher efficiency (>65%) for pharmaceutical contaminants removal as compared to nano-filtration (NF), photo-Fenton, and ozonation granular [104]. Adsorption using granular activated carbon (GAC) is widely adopted as an effective method for the removal of a broad range of contaminants. However, removal efficiency can vary significantly depending on the nature and concentration of pollutants, carbon properties, contact time, and solution chemistry [105]. A combination of AC (granular), sand filter, and ozonation shows a pollutant removal efficiency of 87–95% [106]. Intensive research is being carried out in this field to investigate novel adsorbents in order to improve adsorption capacity and efficiency.

#### 4.1.2. Membrane Filtration

**Figure 3:**
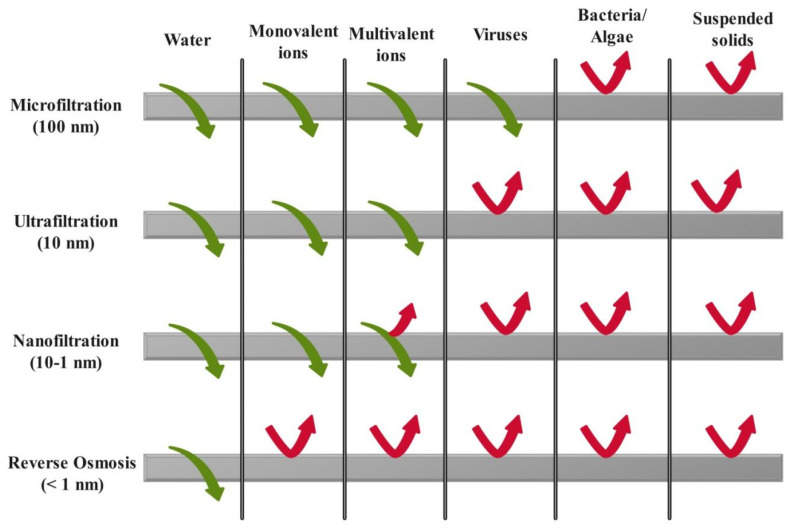
Decontamination capability of different membrane filtration processes.

Membrane filtration, such as reverse osmosis (RO), ultra-filtration (UF), micro-filtration (MF), and nano-filtration (NF), is widely adopted for treatment of pharmaceutical waste. These techniques vary in the pore sizes of the membrane used. MF has the largest pores (0.1–10 microns), followed by UF (0.01–0.1 microns), and then NF with the smallest pores (0.001–0.01 microns). It was found that pharmaceutical contaminants can be removed using both UF and NF. However, the type of filtration technique can be decided on the basis of the target pollutant. MF is suitable for the removal of bacteria and viruses, while NF is applied to clean divalent salts and pesticides [37,107]. Sometimes, the membrane pore size is greater than the effluent size, which makes the membrane permeable to some minor contaminants. In addition to this, membrane recyclability and concentration post-treatment are also major issues with these techniques [108]. The pore sizes of various membrane techniques and their filtration capabilities are shown in Figure 3.

Membrane filtration processes are able to effectively separate a broad range of contaminants. However, their practical implementation is often limited by several operational challenges. Key challenges include fouling, energy requirement, and management of the retentate stream [109]. All types of membrane processes suffer from fouling, which is mainly caused by the physical and chemical interactions between the membrane surfaces and foulants, e.g., microorganisms, ions, organic matter, colloids, or a mixture thereof. Inorganic fouling (scaling) is not a significant fouling contributor as the membranes are typically porous. Membrane fouling can significantly reduce permeate flux and membrane lifespan, necessitating frequent cleaning and increased operational cost. In addition to this, pressure-driven membrane processes, e.g., nanofiltration and reverse osmosis, require substantial energy input, which impacts their overall cost effectiveness and sustainability [32].

Energy consumption in membrane processes majorly depends upon feed water characteristics, operating pressure, and fouling tendency. Another major issue is the management of the concentrated retentate stream. Depending on the feed composition and the type of membrane system used, the retentate may contain high levels of organic pollutants, salts, nutrients, or emerging contaminants, which may require further treatment or safe disposal to prevent secondary pollution. Common management strategies include additional treatment using biological or physico-chemical methods, recirculation to upstream processes, evaporation, or controlled discharge in compliance with environmental regulations [110,111].

##### Nano-Filtration (NF)

Nano-filtration is used for the removal of micro-pollutants. It utilizes pressure gradient as a driving force [112,113]. Nano-filtration removes the pharmaceutical effluents using sieving, electrostatic repulsion, and adsorption. Elimination efficiency depends upon the solubility, hydrophobicity, diffusivity, and polarity of the micropollutants. Other factors can be membrane permeability, pore size, and operating conditions (varying fluxes, rejections/recoveries, trans-membrane pressure, and water feed quality). The performance of NF also depends on the type of membrane used. The water flux and removal efficiency are governed by pore size, membrane material and surface charge. Membrane material, preparation method, and structural configuration collectively decide the overall performance of the NF membrane. Polymeric membranes are the most commonly used thin-film composite (TFC) membranes. Their multilayer structure comprises an ultra-thin polyamide selective layer supported by a porous substrate. TFC membranes exhibit high water permeability combined with excellent solute rejection, making them particularly suitable for complex wastewater matrices such as pharmaceutical effluents. However, the permeability-selectivity tradeoff, membrane fouling, and poor chemical stability in a harsh chemical environment are three major challenges faced by TFC membranes currently [114].

TFC membranes play a critical role in the removal of trace organic contaminants, including active pharmaceutical ingredients, antibiotics, hormones, and their transformation products. The performance of the TFC membrane depends upon physicochemical properties of the PA selective layer, e.g., pore size distribution, surface charge, and hydrophilicity. These properties can be adjusted during membrane fabrication, in accordance with the target pollutant [115,116]. The desirable characteristics of TFC membranes for pharmaceutical wastewater treatment are high rejection efficiency for micropollutants, high water permeability, excellent antifouling properties, chemical stability, and scaling resistance. Thin-film nanocomposite (TFN) membranes and surface functionalization strategies are adopted to enhance antifouling property and chemical resistance. TFN membranes incorporate nanomaterials, e.g., graphene oxide, carbon nanotubes, or metal–organic frameworks into the selective layer and exhibit improved permeability, selectivity, and antifouling properties [117,118,119].

Ceramic membranes overcome this limitation as they are highly resistant to abrasion, fouling and chemical stress, making them ideal for harsh industrial applications. Polyester-based membranes are generally used for dye/salt removal, as these allow monovalent salts to pass. Permeability, selectivity, and fouling resistance of NF membranes may be enhanced by using MXene, cellulose nanocrystals, porous organic polymers, and other emerging nanoparticles [120]. The molecular weight cut-off of NF membranes ranges between 200 and 2000 Da. NF membranes offer specific ion selectivity along with high membrane flux, allowing them to operate at a low operating pressure with high removal efficiency [121,122]. NF is one of the most prominent methods as it is capable of eliminating more than 90% of pharmaceutical contaminants [123]. Xu et al. [124] observed 95.4 and 94.1% removal efficiencies using NF, in the case of indomethacin and sodium diclofenac, respectively. Cuhorka et al. [125] investigated the ibuprofen and diclofenac rejection rates for commercially available NF membranes (AFC 40, AFC 30) and showed that AFC 40 has a 99 and 98.6% rejection rate for ibuprofen and diclofenac, respectively.

##### Microfiltration

High-pressure filtration operation with a micro surface filter has the ability to remove micro-pollutants [126,127]. Microfiltration techniques can be applied individually or may be combined with other suitable treatment options (AOP) [128]. Apart from pharmaceutical waste, microfiltration coupled with the electro-oxidation process can also be used to clean refractory pollutants [129]. Manni et al. [130] developed a low-cost microfiltration system using natural magnesites. The sintering method and uniaxial pressing were used for the fabrication of a flat membrane. Ceramic membranes showed higher thermal resistance along with removal efficiency of 99.9 and 69.7% for turbidity and COD, respectively.

##### Ultrafiltration (UF)

Minimal pressure operation (UF) for waste cleaning has been recommended in the current era [131,132]. Yoon et al. [133] studied the interaction mechanism between 24 EDCs and UF membranes in different water samples, e.g., wastewater, drinking water, etc. The chemistry and source of water were observed to affect the adsorption process. UF suffers from membrane fouling and limited retention ability, leading to low removal efficiency. Kim et al. [95] used UF along with an MOF (metal–organic framework) based adsorbent for the elimination of ibuprofen and 17α-ethinylestradiol. Retention of pharmaceutical compounds was increased, and fouling was reduced as MOF adsorbed the pharmaceutical contaminants. This technique follows an electrostatic and hydrophobic attraction-based mechanism. Singh et al. [134] fabricated UF combined with Cu2O photocatalysts for the removal of Ibuprofen. It showed a removal efficiency of 86% under visible light conditions.

#### 4.1.3. Reverse Osmosis (RO)

RO is a water treatment method used to produce freshwater using pressure difference across a semipermeable membrane [135]. RO consumes more energy and has pharmaceutical compound rejection potential [136]. It has been reported that RO can clean endocrine materials (uncharged) along with other pollutants. Carbamazepine is one of the most common pharmaceutical pollutants, and its removal depends upon the types of membrane used. It is reported that 91 and 85% carbamazepine removal can be achieved by polyamide and cellulose acetate membranes [137]. Licona et al. [112] used a commercial BW30 as a membrane in RO operation to eliminate dipyrone, acetaminophen, diclofenac, caffeine, and ibuprofen and measured the variation in rejection rate with respect to operating pressure and pH of the feed.

#### 4.1.4. Forward Osmosis (FO)

Forward osmosis (FO) is a membrane separation process which is driven by osmotic pressure. FO exhibits better performance as compared to RO, NF, and UF [138,139]. Researchers used FO for the removal of antibiotics from wastewater because it needs less energy and reduces membrane fouling. These advantages were due to the use of an osmotic pressure gradient instead of hydraulic pressure to push the water molecules across the membrane [140]. Pan et al. [141] used a thin-film composite membrane to remove tetracycline from water. 99% tetracycline removal was achieved with low membrane fouling. Samsami et al. [142] designed a thin film FO membrane (nanocomposite) using MIL-53 (Al), a metal–organic framework, to separate doxycycline (DOX) from solution. A removal efficiency of 98.5% was achieved. Generally, FO has a lower water flux than other membrane processes [143,144]. The slower rate of water movement can affect water throughput. Recovery and regeneration of draw solution requires more energy input, which affects the efficiency of the process, and other drawbacks can be membrane scaling and fouling [7,145]

### 4.2. Biological Processes for Removal of Pharmaceutical Contaminants

#### 4.2.1. Aerobic and Anaerobic Treatment

Aerobic and anaerobic methods are considered economical options to treat pharmaceutical waste [10,146]. Mukesh et al. [147] found biological treatment techniques more suitable than physical and chemical techniques for treating pharmaceutical wastes that contain organic impurities [148]. Several researchers have studied the breakdown of pharmaceutical compounds using various bacteria. This can be used as an initial treatment option to clean pharmaceutical waste [149]. Sludge (aerobic, granular, and activated) consists of bacteria that are efficient in treating municipal wastewater [150]. Aerobic treatment is performed by considering bacterial growth in the presence of oxygen by consuming water pollutants, while anaerobic treatment is performed in the absence of air [10]. Generally, aerobic treatment shows rapid pollutant reduction as compared to anaerobic treatment. Moreover, biodegradation and sorption are the main mechanisms used in aerobic techniques [151].

Anaerobic wastewater treatment techniques have numerous benefits, e.g., low surface area, cost-effectiveness, minimal sludge production, high organic loading, sustainability, and most importantly, effectiveness in degrading pharmaceutical contaminants [69,152]. In addition, anaerobic methods reduce nitro-based impurities and induce hydrolysis and demethylation, leading to the breakdown of toxic pollutants into simpler molecules. There are two kinds of bacteria involved in the process. The first one converts organic polymer to simpler compounds (monomers) and the second one converts these fragments into gaseous compounds (CH4) [153]. Anaerobic processes decompose refractory pharmaceuticals into aqueous ones. Various anaerobic wastewater treatment techniques are currently in practice, e.g., membrane bioreactor, anaerobic digestor, anaerobic batch reactor (sequencing), and up-flow reactors (sludge blanket) [154].

**Figure 4:**
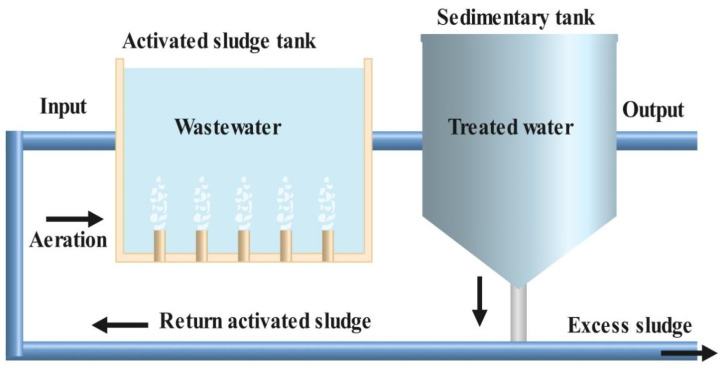
Schematic of the activated sludge process.

The working process of the activated sludge process is shown in Figure 4. In the activated sludge procedure, the organic contaminants act as food for the microorganisms. The microorganism’s growth takes place inside sludge through the metabolization of soluble organic material and then finally combines to form clumped particles. These particles settle down and are further segregated by using a simple settling technique. Activated sludge containing organisms from the last stage of sedimentation is mixed with wastewater [155,156].

An ample amount of air is pumped continuously so as to meet the oxygen demand, and the mixture is continuously stirred to keep the solids suspended. Later on, the mixture is moved to a settling tank (clarifier). In some situations, membrane/flotation tanks can be used to separate microorganisms. Sometimes, partially cleaned water is additionally treated [4,154,157]. Both aerobic and anaerobic treatments can be suitably combined to enhance the overall degradation capability [158]. The hybrid aerobic and anaerobic technique can be the most competent and preferred approach for the treatment of pharmaceutical effluents [159].

#### 4.2.2. Bacteria and Microalgae

Several researchers studied the application of microbes and microalgae for waste cleaning. Endophytes and Pseudomonas are some of the microbes considered in the methods; however, only a few microalgae species may be used to remove pharmaceutical effluents. [159,160]. It is found that a certain community of microbes are capable of cleaning ciprofloxacin, sulfamethoxazole, and 17 β-estradiol in anaerobic conditions [161]. The bacteria identified to degrade ciprofloxacin in nitrate-reducing conditions were from *Comamonas*, *Dysgonomonas*, *Arcobacter*, and *Actinomyces* genera, while the sulfate-reducing community consists of *Desulfovibrio*, *Pepetostreptococcus* and *Enterococcus*.

Park et al. [162] used ammonia-eating microbes to clean pharmaceutical effluents in a membrane bioreactor (MBR). Microalgae are rich in nutrients, possess rapid growth, and can be used in waste cleaning. Microalgae transform waste into lipids/carbohydrates/biooil, etc. [163]. Ding et al. [164] used Navicula sp. to clean atenolol, naproxen, carbamazepine, and ibuprofen with a removal efficiency of 90%. Nayak and Ghosh (2019) [165] used Scenedesmus abundance microalgae to clean pharmaceutical waste in a photobioreactor. These microalgae used pollutants as substrate. Results showed 50% COD, 60–83% nitrate, and phosphate removal.

#### 4.2.3. Constructed Wetland (CW)

**Figure 5:**
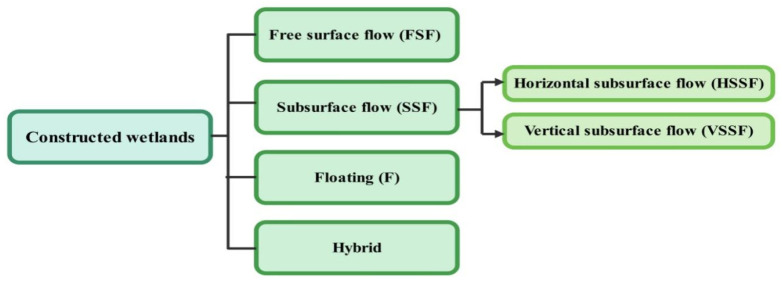
Constructed wetland classification.

Wetlands contain groundwater and flora, which can stay alive in saturated ground situations. Wetlands help in the filtration of polluted waste, groundwater recharging, etc. [60,166]. CW is a cheap and eco-friendly wastewater treatment technique. It forms a semi-aquatic ecosystem that replicates the natural wetland system, leading to a proliferation of organisms and natural vegetation. This initiates various physicochemical reactions leading to wastewater remediation and pollution reduction in nearby water bodies. CW has been classified into FSF (free surface flow), HSSF (horizontal subsurface flow), and VSSF (vertical subsurface flow), floating, and hybrid types, as shown in Figure 5 [167,168,169].

**Figure 6:**
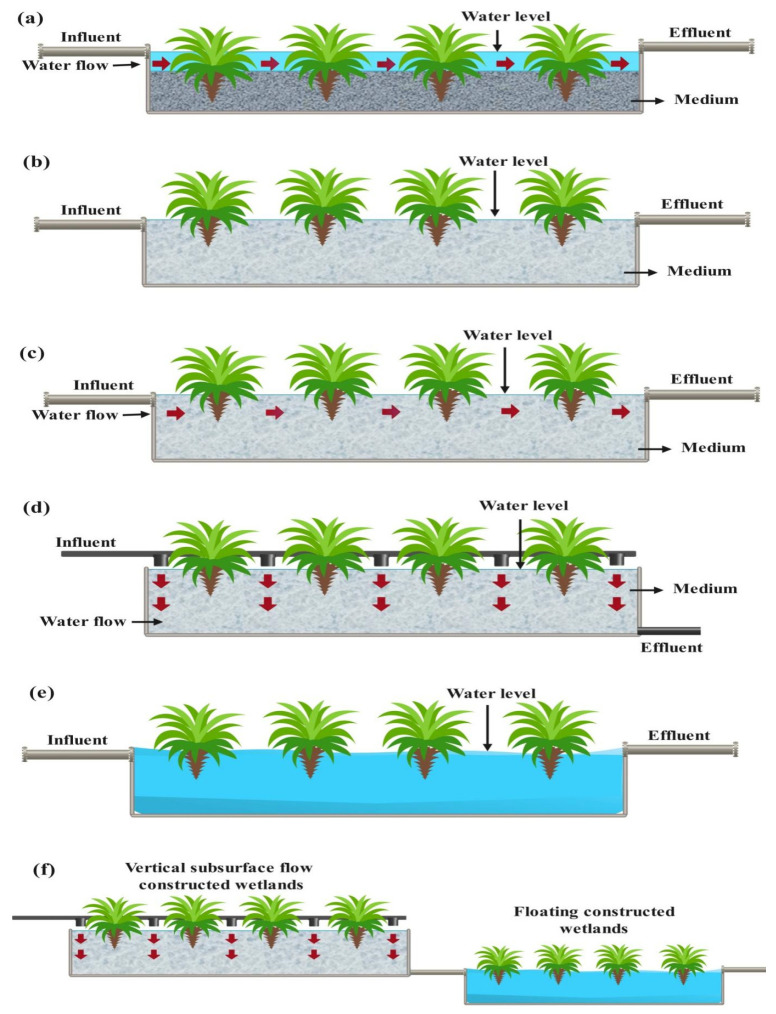
(**a**) FSF type CW, (**b**) SF type CW, (**c**) HSSF type CW, (**d**) VSSF type CW, (**e**) Floating type CW, (**f**) Hybrid type CW.

The dominant sub-processes in CWs are sorption on pollutants, biodegradation, phytoremediation, hydrolysis, adsorption, filtration, plant uptake, photolysis, and photodecomposition [60]. Sharma et al. [167] showed that FSF-based CW is more effective than HSSF-based CW. It is due to more sunlight perception and oxidation, leading to better aerobic biodegradation and photo-degradation. Figure 6a–f show the schematic of various types of CWs.

### 4.3. Advanced Oxidation Process (AOP)

The main mechanism of AOP consists of OH- radical generation, which is capable of oxidizing toxic contaminants from medical, industrial, municipal, and textile wastewater. AOP is suitable for the treatment of pharma waste, mainly due to its enhanced efficacy, low area requirement, and simpler operation [170]. The basic principle involves the generation of OH- radicals, superoxide anions, H_2_O_2_, and other reactive species [171]. AOPs are used in the pharmaceutical industry for decomposition of waste products [172]. The electro-Fenton technique was used by Annabi et al. [173] to study the enoxacin degradation rate.

It has been noticed that AOPs need higher operational and cleaning/maintenance costs as compared to other common techniques [174]. AOP can be classified based on the nature of phases (homogeneous or heterogeneous) or rate and mode of hydroxile formed (photochemical, sonocatalytic, electrochemical, and chemical) [170]. The main reason for degradation using AOPs is the generation of free radicals like superoxide radicals, alkoxyl radicals, and hydroxyl radicals. These radicals further decompose pharmaceutical contaminants into H_2_O and CO_2_ [175]. However, complete mineralization is rarely achieved under practical operating conditions, particularly for complex pharmaceutical compounds. Incomplete oxidation may lead to the formation of transformation products. Some of these transformation products may exhibit higher toxicity, persistence, or biological activity than the parent compounds. For example, ozonation of Carbamazepine produces oxidation by-products with increased ecotoxicological effects. To mitigate these risks, AOPs are often integrated with downstream processes, e.g., biological treatment, adsorption, or membrane filtration (e.g., NF/RO), which can further remove or contain transformation products. Such hybrid treatment strategies are particularly important in pharmaceutical wastewater management to ensure effective, sustainable contaminant removal [176,177].

#### 4.3.1. Photolysis

Conventional biological techniques are applied to treat pharmaceutical waste, but some contaminants (antibiotics/antidepressants) are susceptible to several microbes [178,179]. This necessitates the integration of AOPs such as photolysis downstream of WWTPs [180]. Photolysis, carried out in the presence of ultraviolet rays (UV), leads to the formation of powerful oxidizing agents having the potential to degrade pharmaceutical contaminants [76,175]. Upon exposure to UV or visible light during photolysis, pharmaceutical compounds are promoted to a single-excited state and finally achieve a triplet-excited state.

During this interval, mineralized products are formed either due to the reaction of molecules or the formation of hydroxyl radicals [181]. Visible light and UV rays may be used in combination with hydrogen peroxide or ozone, which produce free radicals to eliminate pharmaceutical contaminants from wastewater [182]. Hora et al. [180] reported degradation of nitrogen-enriched pharmaceutical contaminants using photolysis with UV/visible light. The degradation proceeded via oxidation of hydroxyl radicals, and 47, 50, 60, and 57% degradation was achieved for trimethoprim, carbamazepine, atenolol, and fluoxetine, respectively.

#### 4.3.2. Photo-Fenton

Application of ultra-violet rays (UV) with Fenton is termed as photo-Fenton. In this process, hydrogen peroxide/UV is integrated with Fe-based salts to increase the OH- radical formation during waste sanitization. This technique has the potential to effectively degrade pharmaceutical contaminants present in wastewater [183]. Basically, OH- radical formation depends upon the Fe^3+^ to Fe^2+^ reduction, which can be propelled due to the presence of irradiance. Its efficacy depends on pH, H_2_O_2_ and Fe^2+^ dose and impurities of wastewater [184]. However, hydroxyl radical formation may follow either of two mechanisms. The first method proceeds by reacting Fe^2+^ (Fenton reagent) with H_2_O_2_ to generate Fe^3+^, usually in terms of Fe(OH)^2+^. Further excitement of Fe(OH)^2+^ leads to the production of Fe^2+^ and hydroxyl radical •OH. The second method consists of direct photolysis of H_2_O_2_ to produce •OH [185].

Practically, the iron catalyst cannot be fully recovered or reused efficiently; therefore, continuous dosing of iron salts is required to maintain reaction efficiency in the working pH range (2.5–3). Therefore, a large amount of catalyst is required. Upon pH neutralization after the treatment, a large amount of dissolved iron precipitates as ferric hydroxide, forming sludge. The large amount of sludge creates a crucial operational limitation as it must be managed properly. This also increases the operational cost and may lead to secondary environmental issues if not disposed of properly. These drawbacks can be overcome by using a heterogeneous Fenton process in which iron is immobilized on a suitable solid support, which can be recovered and reused. However, a small amount of sludge formation may take place due to iron leaching [127,186]. This technique was able to decontaminate municipal WWTP effluents containing pharmaceutical contaminants [37,187]. Reactions for the photo-Fenton process are shown below.

(1) $${Fe\left(OH\right)}^{2+}+h\upsilon \to {Fe}^{2+}+OH$$

(2) $$H_{2}O_{2}+h\upsilon \to 2\;OH$$

Dong et al. [188] reported carbamazepine and ibuprofen elimination of 92% with the photo-Fenton mechanism. Overall, it can act as an attractive treatment option for pharma waste removal.

#### 4.3.3. Photocatalysis

The acceleration of a reaction in the presence of a suitable photocatalyst is named photocatalysis. It follows the mechanism of hydroxyl radicals [189,190]. Semiconductors having photocatalytic properties were studied by Kumar et al. [191] for the removal of pharmaceutical contaminants from wastewater. Radicals (hydroxyl) are generated when the waste molecules interact with the catalyst surface. Numerous methodologies, i.e., sol–gel and polymerization, have been adopted for the preparation and reactive surface increment of catalysts to degrade the pharmaceutical contaminants [192].

Photocatalysis involves a lesser number of steps than ozonation and incurs lower operating costs. The process starts with the generation of electron/hole pairs by irradiation of semiconductor catalysts using visible light, known as photogeneration. Further, the transition of photo-generated electrons from the valence band to the conduction band leaves a hole behind. Excited electrons help in the reduction of surface absorbed O_2_ to $O_{2}^{-}$ and holes react with H_2_O to generate ${OH}^{-}$. These radicals have the potential to degrade pharmaceutical contaminants into carbon dioxide and water [191]. The photochemical reactions involved in the process are as follows [37]:

(3) $$Semiconductor+h\upsilon \to e^{-}+h^{+}$$

(4) $$e^{-}+O_{2}\to O_{2}^{-}$$

(5) $$h^{+}+H_{2}O\to H^{+}+OH$$

(6) $$O_{2}^{-}+OH+pharmaceutical\;pollutants\to {CO}_{2}+H_{2}O$$

**Figure 7:**
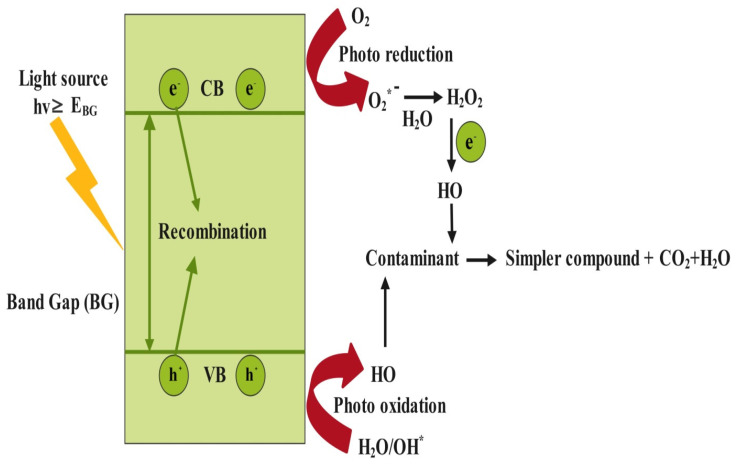
Mechanism of photocatalysis.

A low volume of catalyst lowers the amount of hydroxyl radicals, thus causing inefficient removal of contaminants, while a higher volume could augment turbidity, affecting the transmittance of radiation. Photocatalysts do not undergo any changes during or after the reaction. However, electron-hole pair recombination is a major limitation of this process. If the charge carriers recombine before reacting, the absorbed light energy is wasted as heat or low-energy radiation, resulting in lower radical generation and consequently reduced treatment efficiency. Therefore, a larger volume of reactor and/or longer exposure time may be required. The energy efficiency of the process is also lowered, leading to higher energy demand. A fast recombination rate can degrade the performance of a good photocatalyst, e.g., TiO_2_ [193]. Therefore, controlling charge recombination becomes crucial in photocatalysis. Some common strategies include doping (metal/non-metal), heterojunction formation, electron acceptor addition, and using co-catalysts. Thus, morphological alternation is mandatory to improve and enhance photocatalytic performance [194]. Figure 7 shows the mechanism of photocatalysis.

#### 4.3.4. Ozonation

It is the process of ozone exposure to water using a sparger by bubbling ozone from the bottom of the tank. Direct reaction by ozone leads to oxidation effects and indirect radical generation through a chain of oxidative reactions. Ozone acts as an oxidant for certain organic compounds on its own [195]. The reaction of ozone in the presence of water leads to the formation of hydroxyl radicals [196]. Investigation showed that ozone can affect electron-rich aromatic pharmaceutical compounds, e.g., ciprofloxacin, azithromycin, diclofenac, metoprolol, sulfamethoxazole, carbamazepine, clarithromycin, erythromycin, etc. [30].

**Figure 8:**
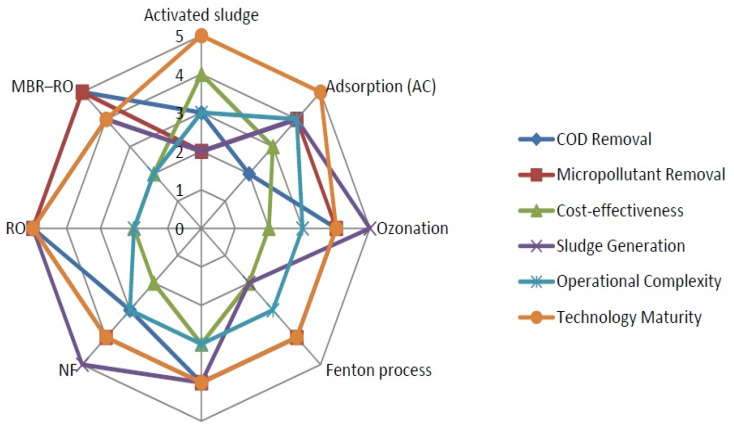
Effectiveness of various treatment options.

Ozone is widely used for the treatment of pharmaceutical contaminants due to its high oxidation potential as compared to other oxidants. However, a higher amount of ozone is required for the complete treatment of organic compounds and other oxidizable substances present in wastewater [197]. It is difficult to store; therefore, it is produced through the most common technique, named the corona discharge method [198]. Another issue with the ozonation process is the production of by-products, which are more toxic than parent compounds [172]. A solution to this issue is the development of hybrid technologies such as ozone/hydrogen peroxide, photocatalytic ozonation, or integration of ozonation with biological treatment processes. Effectiveness of various treatment options (Activated sludge/Adsorption/Ozonation/Fenton/NF/RO and MBR-RO) is shown by a radar plot. The 0–5 scale has been used with 5 parameters, as shown in Figure 8.

### 4.4. Hybrid Processes for Pharmaceutical Contaminants Removal

#### 4.4.1. Hybrid AOP Techniques

There is a need to develop new techniques which can overcome the drawbacks of conventional processes. Individual treatment techniques are not enough for the complete removal of a sufficient amount of pharmaceutical contaminants. Here, ‘complete removal’ refers to concentrations below analytically detectable or environmentally relevant thresholds, rather than absolute elimination. Pharmaceutical residues in aquatic environments are not yet governed by universally harmonized regulatory limits, and permissible concentrations vary significantly. Therefore, treatment performance must be evaluated relative to environmentally relevant thresholds or analytical detection limits. For example, effect-based thresholds for highly potent endocrine-disrupting compounds, such as 17β-estradiol, are typically in the low ng/L to sub ng/L range, indicating their biological activity at extremely low concentrations. Therefore, achieving concentrations below ~1 ng/L should be the target for advanced treatment performance [199].

Treatment up to such low levels generally requires hybrid strategies. Conventional biological systems alone are typically insufficient, whereas advanced hybrid methods, e.g., membrane bioreactors coupled with reverse osmosis, ozonation followed by biological or activated carbon filtration, or hybrid adsorption–membrane systems, have demonstrated the ability to reduce steroid hormones and similar micropollutants to sub-ng/L concentrations under optimized conditions. These approaches combine transformation, adsorption, and physical separation mechanisms, thereby addressing both dissolved and refractory fractions [200]. Hence, a suitable combination of two or more conventional or advanced treatment techniques can be used for the complete or maximum removal of micropollutants or recalcitrant compounds [30]. A combination of photocatalysis and biodegradation has shown exceptional removal efficiency for antibiotics [129].

Hybrid treatment technologies can be systematically classified based on the dominant mechanisms contributing to enhanced performance. These include: (i) biological–membrane systems (e.g., membrane bioreactors combined with nanofiltration or reverse osmosis), where biodegradation reduces bulk organic load and membrane separation removes residual dissolved contaminants; (ii) oxidation–biological systems (e.g., ozonation or Fenton processes followed by biological treatment), where advanced oxidation transforms recalcitrant compounds into more biodegradable intermediates; and (iii) adsorption–membrane or adsorption–oxidation systems, where activated carbon or similar materials concentrate micropollutants and improve subsequent removal efficiency. Hence, the improved performance of hybrid systems arises basically from the complementarity of mechanisms, including transformation, phase transfer, and physical separation, rather than from any single unit process [110,201].

Solar photo-Fenton and adsorption were combined for the removal of flutamide, a well-known anticancer drug, from hospital wastewater. Initially, 20% removal of the drug was achieved using photo-Fenton with 5 mg/L of Fe^+2^ and 50 mg/L of H_2_O_2_. Drug removal increased up to 58% using triple the amount of Fe^+2^ and H_2_O_2_. This pretreated product was subjected to adsorption on activated carbon, which resulted in near complete removal of flutamide [202]. Hou et al. [203] used a combination of an up-flow anaerobic sludge bed and anoxic-oxic tank along with four different AOPs—UV, ozonation, Fenton, and UV/Fenton. Results showed near complete removal of antibiotics through the up-flow anaerobic sludge bed and anoxic-oxic unit. Another research reported the usage of membrane technology and photo-Fenton together, resulting in a shorter treatment time and efficient reagent consumption [204].

The development of membranes having catalytic properties has gained the attention of researchers as they possess several advantages, e.g., better catalytic properties and less fouling. Degradation of cytostatic drugs was achieved using a photocatalytic membrane reactor [205]. Valério et al. [206] investigated the combination of photocatalysis and ozonation techniques for the removal of tetracycline antibiotics. Tetracycline is a widely used, highly persistent antibiotic and exhibits low biodegradability in conventional treatment systems. When treated using a hybrid approach, e.g., photocatalysis combined with ozonation or biological processes, the initial oxidation step breaks down the complex tetracycline structure into smaller, less toxic intermediates. These transformation products are effectively removed through subsequent biological degradation or adsorption steps. Therefore, the hybrid treatment technique results in significantly higher overall treatment efficiency as compared to the individual method. Therefore, A hybrid combination of these two techniques leads to near 100% removal of tetracycline. A combination of a membrane reactor with ozonation was used for the treatment of hospital wastewater [207]. Patel et al. [208] used ozone-based advanced oxidation followed by adsorption using a bed of granular activated carbon for the decontamination of pharmaceutical wastewater. This setup was capable of removing organic compounds, COD, and water quality parameters using ozonation, and the rest of the degraded compounds were removed by adsorption. Sirtori et al. [209] used a photo-Fenton and immobilized biomass reactor sequentially for the removal of nalidix acid and found it to be efficient for the removal of acid.

Ding et al. [164] showed the benefit of UV photolysis before biological treatment for the removal of antibiotics. Different Fenton techniques like dark-Fenton, photo-Fenton, and electro-Fenton were used sequentially with biological treatment techniques by Changotra et al. [210,211] for the treatment of pharmaceutical wastewater, and the result showed that the photo-Fenton process led to a significant reduction in organic load and detoxification, when combined with biological treatment. These results show that a combination of AOPs with biological methods can be used as pre-treatment or post-treatment to biological methods to overcome the drawbacks of individual techniques [212]. A combination of techniques showed better results as compared to individual treatment techniques [213].

The integration of AOPs with adsorption, membrane, and biological treatments demonstrates enhanced overall treatment efficiency in the removal of pharmaceutical contaminants from wastewater. Oxidative processes such as photo-Fenton and ozonation transform persistent and toxic compounds into simpler, more biodegradable intermediates, thereby enhancing the effectiveness of subsequent treatment steps like adsorption or biological degradation. Membrane-based hybrids further improve system performance by ensuring efficient separation and, in some cases, providing catalytic activity that supports simultaneous degradation and filtration.

AOP–biological combinations are particularly effective as pre-treatment strategies for reducing toxicity and organic load, while adsorption and membrane polishing steps are better suited for achieving near-complete removal. These hybrid approaches are especially advantageous for complex waste streams such as hospital and pharmaceutical effluents, where single treatment methods often fall short. However, the selection of an optimum hybrid system depends on several factors, e.g., nature and concentration of contaminant, required effluent quality, operational cost, and system complexity. Hence, hybrid treatment technologies are able to overcome the limitations of individual processes, with AOP-assisted systems emerging as some of the most promising solutions for high-strength and recalcitrant pharmaceutical wastewater.

#### 4.4.2. Hybrid Biological Technology

Membrane bioreactors (MBR) involve a suitable combination of membrane processes and biological processes where the basic principle used for pollutant removal is the physical retention and degradation of microbes on the surface of the membrane. Its efficiency depends upon the physicochemical properties of target compounds, e.g., solute size, charge, molecular weight, geometry, and hydrophobicity. In addition to these, the efficiency also depends upon membrane properties and operating conditions, e.g., porosity, pressure, charge, and pore size [214]. MBR systems are generally effective in removing hydrophobic pharmaceutical compounds due to their tendency to adsorb biomass and undergo biodegradation. However, the removal of hydrophilic compounds is often limited and variable, as these substances exhibit low sorption affinity and are more resistant to biodegradation. On the other hand, pressure-driven membrane processes such as nanofiltration (NF) and reverse osmosis (RO), particularly using thin-film composite (TFC) membranes, are highly effective in removing hydrophilic contaminants through size exclusion and electrostatic interactions [214,215]. Membrane bioreactors (MBRs) integrate biological degradation with membrane filtration, where microorganisms break down organic pollutants while the membrane provides physical separation of biomass and treated effluent. This combination is particularly effective because the membrane retains high concentrations of active biomass, leading to enhanced degradation rates and improved effluent quality, as compared to conventional activated sludge systems. Also, there is no need for secondary clarification and sludge settling due to membrane separation. As a result, MBR systems are especially suitable for treating high-strength or variable wastewater streams where conventional biological systems may not be able to maintain stable performance. Further, MBRs perform well for wastewater containing fine suspended solids or pathogens, as the membrane provides an effective physical barrier. However, higher operational costs are its major limitation, due to membrane fouling and energy requirements. Hence, a suitable combination of these two techniques can be used to remove both the compounds simultaneously and achieve removal efficiency greater than 95% for beta-blockers, EDCs, NSAIDs, hormones, antibiotic resistance genes, and antibiotic resistance bacteria [30]. Wang et al. [216] used MBR in combination with the NF technique to achieve removal efficiency greater than 95% for spiramycin and new spiramycin antibiotics. Such hybrid configurations are particularly well-suited for treating complex pharmaceutical wastewater containing both biodegradable and recalcitrant contaminants. Despite higher operational costs, these systems offer a robust and reliable approach for achieving high-quality effluent, especially in applications requiring stringent discharge or reuse standards. Characteristics of various treatment techniques are presented in Table 2.

#### 4.4.3. Cost Comparison of Various Treatment Methods

The cost comparison for various methods of pharmaceutical wastewater treatment is presented in Table 3. The treatment cost varies between 0.67 and 69 USD/m3 for different treatment options. However, these values are not directly comparable as the treatment cost depends upon several parameters, e.g., the type of contaminants, organic load, operating conditions, etc. [148]. In addition, biological and physical wastewater treatment options require less initial treatment cost as no additional chemicals are required. Conversely, chemical treatment methods need additional chemicals, leading to higher operational costs. Hence, Table 3 signifies chemical treatment options like E-beam + H_2_O_2_, AOP, DIP, etc. require a higher treatment cost compared to many other treatment options. However, less organic load containing wastewater may require fewer chemicals. In this case, the chemical treatment option may need a lower treatment cost.

## 5. Conclusions and Future Scope

The increasing level of pharmaceutical contaminants poses a potential risk to human and animal health as well as the environment. Hybrid treatment systems combining biological processes with advanced polishing steps currently represent the most effective and practically applicable solutions for pharmaceutical wastewater treatment. Configurations such as membrane bioreactor–reverse osmosis (MBR–RO), ozonation followed by biological activated carbon, and adsorption-enhanced biological systems demonstrate high removal efficiencies for bulk organic matter and trace pharmaceuticals, including antibiotics (ciprofloxacin, sulfamethoxazole), analgesics (diclofenac, ibuprofen), and endocrine-disrupting compounds such as 17β-estradiol. Adsorption–biological hybrids, such as powdered activated carbon combined with activated sludge or MBR systems, also provide a cost-effective option for mitigating a broad range of micropollutants. Despite these advantages, removal of certain persistent and polar compounds, e.g., carbamazepine, along with oxidation-derived transformation products, is a major challenge for current hybrid strategies.

Future research should focus on optimizing hybrid system configurations so as to become more effective, especially against persistent pollutants, e.g., sulfamethoxazole, estrone, etc. Particular attention should be given to minimizing energy demand while maintaining high removal efficiency for persistent pharmaceuticals, including carbamazepine, fluoroquinolones (e.g., ciprofloxacin) and anti-inflammatory drugs (e.g., diclofenac). Research should focus on the circular economy framework via a resource-oriented recovery approach. This includes enabling safe water reuse, recovering energy through biological processes, and exploring recovery of valuable compounds. The integration of hybrid systems with low-energy polishing units, renewable energy inputs, and nature-based treatment steps could significantly enhance overall system resilience.

## Figures and Tables

**Table 1:** The removal efficiency of various wastewater treatment techniques.

Technique	Target Pollutant	Initial Concentration	Reaction Time (RT)/Hydraulic Retention Time (HRT)	System Scale	Matrix Type	Remarks	Removal Efficiency	References
**Biological**								
Anaerobic baffled reactor	Ampicillin	3.5 mg/L	2.5 days	Lab-scale	Real wastewater	pH—6.8	42.1%	[69]
Anaerobic baffled reactor	Aureomycin	4.6 mg/L	2.5 days	Lab-scale	Real wastewater	pH—6.6	31.3%	[69]
Activated sludge process	Acetamenofen	7460 ng/L	10 h	Actual treatment plant	Real wastewater	-	99.9%	[70]
Activated sludge process	Naproxen	2584 ng/L	10 h	Actual treatment plant	Real wastewater	-	95.7%	[70]
Aerobic submerged MBR + sponge-plastic biocarriers	Sulfadiazine	5 µg/L	12 h	Lab-scale	Synthetic wastewater	SRT—45 days	91%	[71]
	Sulfamethoxazole	5 µg/L	12 h				88%	
Anaerobic bioreactors	Tetracyclines	300 µg/L	120 h	Lab-scale	Synthetic wastewater	-	>90%	[72]
Biofilm membrane bioreactor	Sulfonamide	8.21	1–2 days	Lab-scale	Real wastewater	-	98.8%	[73]
Fungal membrane bioreactor	Diclofenac	345 ± 110 µg/L	2 days	Lab-scale	Synthetic wastewater	-	55%	[74]
	Bisphenol A	475 ± 25 µg/L	2 days				80–90%	
**AOP**								
UV-A-LED Photo-Fenton	Antipyrine	50 mg/L	60 min	Lab-scale	Synthetic wastewater	-	93%	[75]
UV-C/H_2_O_2_	ciprofloxacin	100 µg/L	60 min	Pilot-scale	Synthetic wastewater		>99	[76]
	Sulfamethoxazole	100 µg/L	60 min				>99	
Sludge Ozonation	Tetracyclines	100 µg/L		Lab-scale	Synthetic wastewater		86.4–93.6%	[77]
	Azithromycin	100 µg/L						
Microbial Electro-Fenton	Tylosin, Tetracycline, Sulfaquinoxaline		24 h	Lab-scale	Synthetic wastewater		93.5 ± 1.3 94.1 ± 1.3 94.4 ± 1.5	[78]
Photocatalysis	Imipenem	500 µg/L	60 min	Lab-scale	River water		75%	[79]
	Meropenem	50 µg/L	45 min				75%	
Ozonation	Ketoprofen	106.8 ng/L	15 min	Pilot-scale	Synthetic wastewater	O_3_—9 mg/L	99%	[80]
	Atenolol	23.9 ng/L	15 min			O_3_—3 mg/L	100%	
	Primidone	21.3 ng/L	15 min			O_3_—9 mg/L	92%	
Ozonation	Tetracycline	50 mg/L	60 min	Lab-scale	Synthetic wastewater		99%	[81]
Fenton oxidation reaction	Sulfonamide	100 µg/L	120 min	Lab-scale	Real wastewater	pH—6.0 H_2_O_2_—2.9 mM	74%	[82]
Ozonation	Sulfonamide	1000 µg/L	35 min	Lab-scale	Synthetic wastewater		> 99.9%	[83]
Heterogeneous Electro-Fenton	Amoxicillin	20 mg/L	60 min	Lab-scale	Synthetic wastewater	nano Fe_3_O_4_ Catalyst	98%	[84]
**Physical**								
Microfiltration Membrane	Triclosan, Acetaminophen Ibuprofen	1 mg/L	120 min	Lab-scale	Synthetic wastewater		10–95%	[85]
Adsorption (Activated Carbon)	Diclofenac	10–50 mg/L.	upto 210 min	Lab-scale	Synthetic wastewater	Adsorption capacity 178.9 mg/g	~99%	[86]
Ultrafiltration Carbon-polymeric membranes	Diclofenac Paracetamol Metronidazole	10 ppm	60 min	Lab-scale	Synthetic wastewater	MC 0.5 * PAC 2.5 **	50.44% 41.57% 36.20%	[87]
Adsorption (Activated Carbon)	Sulfamethoxazole	100 mg/L	120 h	Lab-scale	Synthetic wastewater	Adsorption capacity 417 mg/g	>99.9%	[88]
Adsorption (Metal oxides)	Sodium Diclofenac	50 mg/L	60 min	Lab-scale	Synthetic wastewater	C-Zn	76.5%	[89]
Adsorption (Activated Carbon)	Carbamazepine	50 mg/L	60 min	Lab-scale	Municipal wastewater	Adsorption capacity 182.9 mg/g	93%	[90]
Nanofiltration	Diclofenac Ibuprofen Paracetamol	100 mg/L	60 min	Pilot-scale	Synthetic wastewater	-	99.7% 81.2% 49%	[91]
Adsorption (Granular activated carbon)	17β-estradiol17α ethinylestradiol Bisphenol A	100 mg/L	60 min	Pilot-scale	Synthetic wastewater	Adsorption capacity 4.01 mg/g 2.97 mg/g 16.26 mg/g	96.98% 97.05% 96.21%	[92]
Adsorption (Tea leaves)	17β-estradiol17α ethinylestradiol Bisphenol A	100 mg/L	60 min	Pilot-scale	Synthetic wastewater	Adsorption capacity 3.46 mg/g 2.44 mg/g 18.35 mg/g	95.75%, 95.25% 96.19%	[92]
Reverse osmosis	Ciprofloxacin	500 µg/L	180 min	Pilot-scale	Synthetic wastewater		99.7%	[93]
		200 µg/L					98.3%	
		50 µg/L					97.8%	
**Hybrid**								
Photocatalytic membrane (T-PS)	Diclofenac	10 µM	24 h	Pilot-scale	Synthetic wastewater		93%	[94]
	17α ethinylestradiol	10 µM	24 h				96%	
Metal–organic frameworks with ultrafiltration hybrid systems (MOF-UF)	Ibuprofen	50 mg/L	2 h	Pilot-scale	Synthetic wastewater	pH—11	57.9%	[95]
	17α ethinylestradiol	50 mg/L	2 h				72.2%	
Forward osmosis membrane bioreactor	Carbamazepine	50 µg/L	9 h	Lab-scale	Synthetic wastewater		88.20–94.45%	[23]
		100 µg/L						
		200 µg/L						
Photocatalytic membrane reactor	Diclofenac	0.12, 0.61 and 8.85 mg/L	30 min	Pilot-scale	Real wastewater		56–100%	[96]
			60 min					

* MC—Methylcellulose; ** PAC—Powdered activated carbon.

**Table 2:** Characteristics of various treatment techniques.

Treatment Category	Process	Mechanism/Role in Removal	Advantages	Limitations	Practical Applicability	References
**Physical**	Activated carbon adsorption	Physical adsorption of hydrophobic and moderately polar pharmaceuticals via surface interaction	High removal efficiency for many pharmaceuticals; simple operation; widely available	Saturation and regeneration requirements; high operational cost for spent carbon handling	Effective as a polishing step for antibiotics, hormones and analgesics	[217]
**Chemical**	Chemical oxidation	Chemical transformation of organics into smaller/less toxic compounds	Applicable to a broad contaminant spectrum; improves biodegradability	Sludge/by-product formation; chemical consumption; incomplete mineralization risk	Suitable as a pre-treatment or partial oxidation step	[217]
	Coagulation– precipitation	Charge neutralization and floc formation for particulate-bound contaminants	Low capital cost; simple operation; fast kinetics	Less effective for dissolved micropollutants; sludge management issue	Primarily for turbidity, colloids, and partial COD reduction	[217,218]
	Sodium hypochlorite oxidation	Oxidative chlorination of organic compounds	Effective disinfection; partial oxidation of organics	Formation of toxic chlorinated by-products; high chemical cost	Limited use due to environmental concerns	[219]
**Biological**	Aerobic treatment (activated sludge)	Microbial biodegradation under oxygen-rich conditions	Effective for bulk COD and colour removal; well-established	Poor removal of recalcitrant pharmaceuticals; long HRT	Core municipal and industrial treatment step	[217]
	Anaerobic treatment	Anaerobic biodegradation producing biogas	Energy recovery potential; tolerant to high-strength wastewater	Long start-up/acclimation; limited micropollutant degradation	Suitable for high organic load pre-treatment	[220]
**Advanced oxidation processes (AOPs)**	Ozonation	Oxidative breakdown via ozone and radical formation	No sludge generation; effective for many micropollutants	Short ozone half-life; energy demand; incomplete mineralization possible	Strong polishing step for pharmaceuticals and endocrine disruptors	[221]
	Irradiation (UV/ionizing)	Radical generation via high-energy radiation	Broad-spectrum oxidation; effective degradation of resistant compounds	High energy demand; scalability limitations	Mostly pilot-scale or specialized applications	[221]
	Photocatalysis	Light-activated catalyst generates reactive radicals	Simultaneous removal of organic/inorganic pollutants; low secondary waste	Slow kinetics; limited large-scale implementation	Emerging technology for niche applications	[222]

**Table 3:** Cost comparison of various treatment techniques.

Method	COD Reduction (%)	Total Treatment Cost (USD/m^3^)	Reference
AOP	40–50	22.46	[148]
AOP + RBC	20–45	14.00	[148]
DIP	60–80	59.00	[148]
Catalytic wet air oxidation	70	6.67	[223]
Fenton	99.7	0.852	[223]
Photo-Fenton	95	10.36	[223]
E-beam	48.1	0.67	[224]
Activated sludge	60.1	0.70	[224]
E-beam + H_2_O_2_	89	11.48	[224]
Membrane bioreactor	>99.5	37.7–69.5	[225]
Direct contact membrane distillation	80	1.37	[226]
Nanofiltration	70	0.63	[226,227]
Coagulation precipitation + MBR	96.6–98.3	0.25	[201]
Hydrolytic acidification + A/O + MBR	87.5–91.6	0.11	[201]
UF+ RO+ triple effect evaporation	>99.5	5.51–6.48	[201]
Biochemical treatment + UF+ RO+ ion exchange	>90%	0.63	[201]

## Data Availability

The original contributions presented in the study are included in the article, further inquiries can be directed to the corresponding authors.

## Abbreviations

The following abbreviations are used in this manuscript:

AC	Activated carbon
AOP	Advanced oxidation process
BOD	Biological oxygen demand
COD	Chemical oxygen demand
CW	Constructed wetland
EDC	Endocrine-disrupting compounds
FO	Forward osmosis
MBR	Membrane bioreactor
MF	Microfiltration
MOF	Metal–organic framework
NF	Nanofiltration
NSAID	Nonsteroidal anti-inflammatory drugs
RBC	Rotating biological contactor
RO	Reverse osmosis
UF	Ultrafiltration
WWTP	Wastewater treatment plant
